# Antisecretory Factor May Reduce ICP in Severe TBI—A Case Series

**DOI:** 10.3389/fneur.2020.00095

**Published:** 2020-03-06

**Authors:** David Cederberg, Hans-Arne Hansson, Edward Visse, Peter Siesjö

**Affiliations:** ^1^Department of Neurosurgery, Skane University Hospital, Lund, Sweden; ^2^Institute of Biomedicine, University of Gothenburg, Gothenburg, Sweden

**Keywords:** traumatic brain injury, intracranial hypertension, therapeutic agents per oral treatment, ICP reduction, anti-inflammatory therapy, novel treatments against traumatic brain edema

## Abstract

Traumatic brain injury (TBI) constitutes a global epidemic. Overall outcome is poor, with mortality ranging from 10 to 70% and significant long-term morbidity. Several experimental reports have claimed effect on traumatic edema, but all clinical trials have failed. Antisecretory factor, an endogenous protein, is commercially available as Salovum®, which is classified as a medical food by the European Union and has been proven effective in experimental trauma models. It has, however, previously not been tested in humans with severe TBI. We hereby report a case series of five adult patients with severe TBI, treated with Salovum. The objective of the intervention was to evaluate safety and, if possible, its effect on intracranial pressure and outcome. Patients received 1 g Salovum per kilo of body weight divided into six doses per 24 h. Each dose was administered through the nasogastric tube. Patients were scheduled for 5 days of treatment with Salovum. Intracranial pressure was controlled in all patients. In three of five patients, intracranial pressure could be controlled with Salovum and deep sedation (no barbiturates), except during periods of gastroparesis. Five of five patients had a favorable short-term outcome, and four of five patients had a favorable long-term outcome. No toxicity was observed. We conclude that at least three of the five treated patients experienced an effect of Salovum with signs of reduction of intracranial pressure and signs of clinical benefit. In order to validate the potential of antisecretory factor in TBI, a prospective, randomized, double-blind, placebo-controlled trial with Salovum has been initiated. Primary outcome for the trial is 30-day mortality; secondary outcomes are treatment intensity level, intracranial pressure, and number of days at the neurointensive care unit.

## Introduction

Traumatic brain injury (TBI) constitutes a global burden despite the fact that mortality and morbidity have been reduced in several countries during the last decades (1, 2). Advances in neurointensive care, cerebral monitoring, and neuroradiology have improved outcome for patients with severe TBI, but the results globally are still poor with a mortality ranging from 10 to 70% and significant long-term morbidity (3).

Traumatic brain injury encompasses several pathogenic mechanisms as primary mechanical injury and hemorrhage followed by secondary events such as vasospasm, inflammation, excitotoxic cell damage, and energy deprivation but also long-term progressive brain tissue degeneration. One common denominator in TBI is cerebral edema, which may cause raised intracranial pressure (ICP) and is a major factor responsible for mortality and morbidity in TBI (4). The pathophysiologic mechanisms of cerebral edema are, however, only partially known (5).

Although several experimental reports have claimed effect on traumatic cerebral edema, all clinical trials have failed (6).

Antisecretory factor (AF) is a 41-kDa endogenous protein proposed to possess both antisecretory and anti-inflammatory effects (7). The exact mechanism of AF is unknown, but it has been proposed to act by modulation of proteasomes, complement, and myeloid cells (8–10). A recent report shows that AF inhibits the NKCC1 ion pump; the latter also has been implicated in the evolution of edema in TBI (11, 12).

Salovum® is an egg yolk powder enriched for AF and classified as food for specific medical purposes in the EU. Salovum has been used in clinical trials for gastroenteritis and Ménière inflammatory bowel disease, and no toxicity has been reported [Lantmännen Functional Foods AB Besöksadress: S:t Göransgatan 160, Stockholm, Sweden, (13)].

The functional part of AF has been synthesized within a 16-amino-acid peptide, AF16. AF16 and AF have shown effects against cerebral edema and increased ICP in models of herpes encephalitis and TBI (14, 15).

We hereby report the first five patients with severe TBI, treated with the AF-enriched dietary supplement Salovum with the aim to assess ICP control and clinical outcome.

## Patients and Methods

### Patients

Patients with severe TBI (Glasgow Coma Scale score <9) were admitted to the neurointensive care unit (NICU), Skåne University Hospital. The treated patients were recruited during 2015 and 2017. Follow-up data were collected in December 2018.

### ICP Monitoring

All patients were monitored for ICP, using Spiegelberg™ tunneled parenchymal probes placed in the right frontal lobe through a separate burr hole.

### Treatment Algorithm

Patients were treated according to the local treatment algorithm for TBI, also known as the Lund Concept (LC) (16, 17). Five patients where first-tier treatment with LC did not control ICP were treated with Salovum after consent from next of kin. See Appendix (S1).

### Treatment Intensity Level

Treatment intensity level (TIL) was used to display the measures taken to control ICP during treatment (18).

### Ethical Permission

Ethical permission was granted by the regional ethical review board of Lund University, no. 2013/144.

### Administration of Salovum

The patients received 1 g Salovum per kilo of body weight divided into six doses per 24 h. Each dose was mixed with 100 mL of water and administered through the indwelling nasogastric tube. Patients were scheduled for 5 days of treatment with Salovum. The dosage of Salovum and the dosage interval were chosen from the lower ranges of dosages as reported from prior human trials with Salovum (19).

### Control of Gastroparesis

There is no simple way to measure how much AF is delivered to the patient. Because of these circumstances, we can merely conclude when we almost certainly know when no AF was delivered.

Salovum was given every 4 h via the nasogastric tube. Gastroparesis was suspected prior to the administration, if the volume acquired by aspiration via the tube was more than 150 mL.

## Results

General patient characteristics are summarized in Table 1. During Salovum treatment, ICP >25 mm Hg occurs in 0–11% of hourly measurements. After 24 h with Salovum, ICP >20 mm Hg occurs in 3–33% of hourly measurements. Gastroparesis (gastric fluid volume >150 mL) is suspected in 0–28% of measurements.

**Table 1:** Summary of patient characteristics and interventions.

**Patient no**	**1**	**2**	**3**	**4**	**5**
**Age/sex**	**13/male**	**35/male**	**16/male**	**56/male**	**63/male**
**Injury type**	**No mass lesion**	**Contusion SAH**	**Contusion SAH**	**ASDH contusion**	**ASDH contusion**
^a^GCS	3	7	6	7	5
^b^Pupils	–/–	–/–	–/–	–/–	+/–
Trauma	^c^mv	mv	mv	mv	fall
Barbiturates	Y	Y	N	N	N
Non ^d^DC surgery	N	Y	N	Y	N
DC	N	Y	N	N	N
^e^GOSE	7	^f^1	7	6	6
^g^ICP>25 (%)	11	6	0	6	4
^h^ICP>20 (%)	27	22	3	33	31
^i^Gastroparesis (%)	4	28	0	8	10

a *Glasgow Coma Scale*.

b On admission.

c Motor vehicle.

d Decompressive craniectomy.

e Glasgow outcome scale extended.

f Death after hemorrhage under scalp after gaining consciousness.

g Percent of ICP measurements >25 mm Hg during Salovum treatment.

h Percent of ICP measurements >20 mm Hg after 24 h during Salovum treatment.

i *Percent of gastric retention measurements >150 ml during Salovum treatment*.

### Patient 1

**Figure 1:**
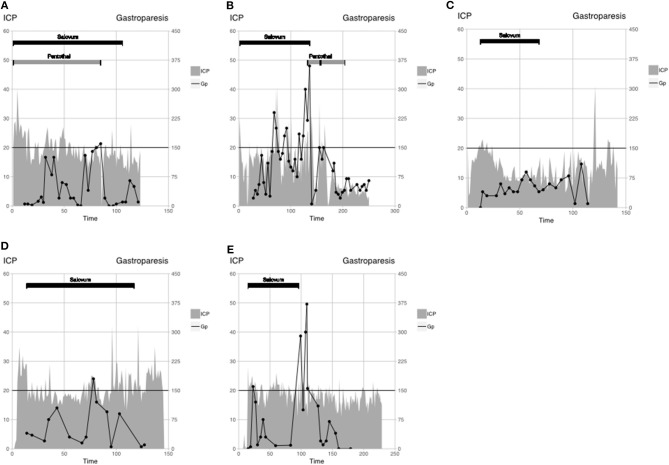
Patient monitoring data are displayed as ICP (gray-filled line), gastroparesis (black line), and interventions: Salovum (black bar), Pentothal (gray bar). Critical ICP threshold according to the TBI algorithm (20 mm Hg) and threshold for gastroparesis are shown by horizontal black line. **(A)** patient 1, **(B)** patient 2, **(C)** patient 3, **(D)** patient 4, and **(E)** patient 5.

A 13-year-old boy was involved in a bicycle accident. No mass lesion was seen on the initial computed tomography (CT). Because of uncontrollable ICP with first-tier treatment according to LC, barbiturates and Salovum were administered (Figure 1A). Intracranial pressure decreases to <20 mm Hg within 12 h, but during periods of gastroparesis, ICP increases to >20 mm Hg. Barbiturates could be discontinued, and ICP remains low. Treatment intensity level score was between 6 and 9. Salovum was administered during 4 days (107 h). Intracranial pressure >25 mm Hg occurs in 10 of 107 hourly measurements (11%) during Salovum treatment. After 24 h of treatment, ICP >20 mm Hg occurs in 23 of 84 of hourly measurements (27%). Gastroparesis is suspected in 2 of 26 measurements. At follow-up, the patient is Glasgow Outcome Scale–Extended (GOSE) (2).

### Patient 2

A 35-year-old man was involved in a bicycle accident, with small frontal contusions and traumatic subarachnoid hemorrhage on initial CT. Because of uncontrollable ICP, using first-tier treatment of LC, Salovum is administered (Figure 1B). Twenty-four hours after admission, one of the patient's frontal contusions has greatly expanded and is surgically evacuated despite ICP of <20 mm Hg. On day 3 (71 h) after admission, CT shows progress of numerous contusions. One contusion is surgically evacuated, and the patient receives a bilateral hemicraniectomy. After surgery, ICP is controlled until day 5 (141 h), when gastroparesis recurs. Salovum is discontinued, and barbiturate treatment is initiated whereby ICP can be controlled during the rest of the NICU period. Treatment intensity level score was between 9 and 14. Salovum is given during 5 days (137 h). Intracranial pressure >25 mm Hg occurs in 8 of 137 hourly measurements (6%) during Salovum treatment. After 24 h of treatment, ICP >20 mm Hg occurs in 22 of 103 hourly measurements (21%). Gastroparesis is suspected in 4 of 14 measurements. The patient wakes up and has GCS of 14 but later goes ad mortem due to an unexpected extracerebral bleeding under the scalp flap.

### Patient 3

A male patient younger than 18 years was involved in a motor vehicle accident. Initial CT shows traumatic subarachnoid hemorrhage, obliterated basal cisterns, and small hemorrhagic contusions. Because of uncontrollable ICP using the first-tier treatment of LC (>30 mm Hg between the sampling times), a new CT scan was obtained, which shows no progression of the contusions, but slightly increased edema. Salovum is administered 13 h after admission (Figure 1C). Almost immediately, a decrease in ICP can be observed, and ICP is thereafter controlled. No other ICP-lowering measures were taken. Treatment intensity level score was between 1 and 3. Salovum was given during 3 days (55 h). Intracranial pressure >25 mm Hg occurs in none of 55 hourly measurements during Salovum treatment. After 24 h of treatment, ICP >20 mm Hg occurs in 1 of 31 of hourly measurements (3%). Gastroparesis is suspected in none of 14 measurements. At follow-up, the patient is GOSE 7.

### Patient 4

A 56-year-old man was involved in a moped accident, where initial CT shows an acute subdural hematoma and numerous small contusions. The subdural hematoma is evacuated. Postoperatively, ICP is uncontrollable using first-tier treatment of LC. Salovum is administered 13 h after admission (Figure 1D). No other ICP-lowering measures were taken. Except for a brief period of gastroparesis, ICP is under control. Treatment intensity level score was between 2 and 3. Salovum is given during 5 days (103 h). Intracranial pressure >25 mm Hg occurs in 6 of 103 hourly measurements (6%) during Salovum treatment. After 24 h of treatment, ICP >20 mm Hg occurs in 26 of 79 hourly measurements (33%). Gastroparesis is suspected in 1 of 13 measurements. At follow-up, the patient is GOSE 6.

### Patient 5

A 63-year-old man was involved in a falling accident. Initial CT shows an acute subdural hematoma and numerous middle-sized contusions. The subdural hematoma is evacuated. Salovum is administered 15 h after admission (Figure 1E). Because of high ICP, several new CT scans are performed, revealing no progression of the contusions. Intracranial pressure is kept at <25 mm Hg at all times; no further surgery is needed. On day 5, gastroparesis is diagnosed with an ensuing slight elevation of ICP. When gastroparesis is attenuated, ICP returns to normal. Treatment intensity level score was between 2 and 3. Salovum is given during 3 days (81 h). Intracranial pressure >25 mm Hg occurs in 3 of 81 hourly measurements (4%) during Salovum treatment. After 24 h of treatment, ICP >20 mm Hg occurs in 18 of 57 hourly measurements (31%). Gastroparesis is suspected in 1 of 10 measurements. At follow-up, the patient is GOSE 6.

## Discussion

The present case study is the first publication on treatment of severe TBI with AF. We conclude that at least three of five treated patients may have experienced a beneficial effect of Salovum with reduction of ICP and very low TIL scores, since few other ICP-lowering measures were made.

The LC treatment algorithm is very restrictive in terms of which interventions are readily utilized. Lund Concept uses a physiology-oriented approach in which the patients' cerebral perfusion pressure is kept at low levels compared to traditional guideline recommendations. By doing so, it is hypothesized that the development of brain edema is kept at a minimum. Development of secondary injury is thought to be avoided partly because of less edema, partly by optimizing circulation physiologically and avoiding vasopressors that may compromise microcirculation. See Appendix.

The TIL score for LC generates low values, even when second-tier therapies are initiated (maximum 12/38). See Appendix.

Salovum is an egg powder enriched for AF but with no specified quantity or concentration of the protein. The delivered amount of AF could thus vary among the treated patients.

The uptake of AF after oral administration is, as for most oral drugs, dependent on functioning gastrointestinal passage. Gastroparesis is common in patients treated according to the LC, because of high doses of fentanyl. Gastroventricular fluid volume is not the gold standard for diagnosing gastroparesis, but a gastric fluid volume of 100–150 mL indicates decreased gastric emptying and therefore decreased uptake of oral drugs. In three of the patients, ICP was reduced during administration of Salovum as the only ICP-lowering measure taken. We could see that the effect was attenuated at periods with suspected gastroparesis.

All patients had good outcomes as assessed by GOSE at 6 months, with the exception of one patient who died of a very unusual complication, which was most probably related to the preceding decompressive craniotomy. This patient did, however, wake up to a GCS score of 14, indicating a good prognosis.

Treatment of patients with severe TBI by the LC has reported a reduction of mortality and morbidity in selected patients, but overall mortality and morbidity for all patients admitted with severe TBI are still considerable (unpublished data). The patients included in this report had an uncontrollable ICP despite application of the treatment algorithm's first-tier treatments. The first two patients were administered pentothal and Salovum because of the uncertainty of the effect of the latter, whereas the three ensuing patients were given only Salovum and did not need any other ICP-lowering treatment. As in previously reported clinical trials, we could not see any obvious toxicity in the current case series.

Although the present results do not prove that AF can reduce ICP in TBI, and thus improve outcome, there are strong implications for this. The patients had all been treated according to the first tier of the LC TBI algorithm, currently a modification of the original LC, but despite this, ICP could not be controlled until Salovum treatment was initiated. The LC treatment algorithm allows for relatively few ICP-lowering measures according to the TIL score. Second-tier treatment options were utilized in only two of these patients, which means that numerous other actions could have been taken and proven equally effective. However, it appears that Salovum might be an effective agent in controlling ICP and with no observed toxicity. In order to validate the potential of antisecretory factor in TBI, a prospective, randomized, double-blind, placebo-controlled trial with Salovum in severe TBI has been initiated (NCT03339505). Primary outcome for the trial is 30-day mortality; secondary outcomes are TIL, ICP, and number of days at the NICU.

## Data Availability Statement

The datasets generated for this study are available on request to the corresponding author.

## Ethics Statement

The studies involving human participants were reviewed and approved by Regional ethical review board of Lund University, LU, nr 2013/144. Written informed consent to participate in this study was provided by the participants' legal guardian/next of kin.

## Author Contributions

All authors listed have made a substantial, direct and intellectual contribution to the work, and approved it for publication.

### Conflict of Interest

The authors declare that the research was conducted in the absence of any commercial or financial relationships that could be construed as a potential conflict of interest.
