# Supplementary material for: Antisecretory Factor May Reduce ICP in Severe TBI—A Case Series
Source: Front Neurol. 2020 Mar 6;11:95. doi: 10.3389/fneur.2020.00095 (PMC7067821; doi:10.3389/fneur.2020.00095)
Supplement: Supplementary file 1 [file Data_Sheet_1.PDF]

Lund Concept Treatment Algorithm

First-Tier Therapy

		TIL score
Intubation		
	Normoventilation	
Deep sedation and analgesics		
	Midazolam	*2*
	Fentanyl	
Normovolemia		
	Albumin	
	Blood	
Anti-hypertensives		
	Metoprolol	
	Clonidine	
Head elevation		
	30° maximum	*1*

Second-Tier therapy

CSF-drainage		
	low volume (<5 ml/h)	*2*
Fluid loading for maintaining CPP		
	Albumin 20%	*1*
Barbiturate coma		
	Burst supression	*5*
Vasopressor therapy for maintaining CPP		
	Low dose Dobutamine	*1*

Third-Tier therapy

Decompressive craniectomy/2:nd surgery	4 - 5
